# How do patient and hospital features influence outcomes in small-cell lung cancer in England?

**DOI:** 10.1038/bjc.2011.310

**Published:** 2011-08-09

**Authors:** A L Rich, L J Tata, C M Free, R A Stanley, M D Peake, D R Baldwin, R B Hubbard

**Affiliations:** 1 Division of Epidemiology and Public Health, University of Nottingham, Hucknall Road, Nottingham, NG5 1PB, England; 2 Department of Respiratory Medicine, University Hospitals of Leicester, Glenfield Hospital, Groby Road, Leicester, LE3 9QP, England; 3 National Health Service Information Centre for Health and Social Care, 1, Trevelyan Square, Leeds, LS1 6AE, England; 4 Department of Respiratory Medicine, Nottingham University Hospitals, City campus, Hucknall Road, Nottingham, NG5 1PB, England

**Keywords:** small-cell lung cancer, chemotherapy, survival, epidemiology

## Abstract

**Background:**

Our aim was to systematically determine how features of patients and hospitals influence access to chemotherapy and survival for people with small-cell lung cancer in England.

**Methods:**

We linked the National Lung Cancer Audit and Hospital Episode Statistics and used multiple logistic and Cox regression analyses to assess the influence of patient and hospital features on small-cell lung cancer outcomes.

**Results:**

There were 7845 patients with histologically proven small-cell lung cancer. Sixty-one percent (4820) of the patients received chemotherapy. Increasing age, worsening performance status, extensive stage and greater comorbidity all reduced the likelihood of receiving chemotherapy. There was wide variation in access to chemotherapy between hospitals in general and patients first seen in centres with a strong interest in clinical trials had a higher odds of receiving chemotherapy (adjusted odds ratio 1.42, 95% confidence interval (CI) 1.06, 1.90). Chemotherapy was associated with a lower mortality rate (adjusted hazard ratio 0.51, 95% CI 0.46, 0.56).

**Conclusion:**

Patients first seen at a hospital with a keen interest in clinical trials are more likely to receive chemotherapy, and chemotherapy was associated with improved survival.

Chemotherapy is recommended by the National Institute for Health and Clinical Excellence for the treatment of individuals with small-cell lung cancer (National Institute for Clinical Excellence, 2005), but there is evidence that geographical variation exists in its use across England (National Lung Cancer Audit, 2009). The extent to which this variation is due to patient features, including comorbidity and performance status, or features of the hospital where the patient is first seen, is not known; and establishing this is a priority given the poor survival for people with lung cancer seen in the United Kingdom (Verdecchia *et al*, 2007; Coleman *et al*, 2011).

We have used the recently validated National Lung Cancer Audit (NLCA) dataset (Rich *et al*, 2011b) together with comorbidity data from Hospital Episode Statistics to study the impact of patient features and features of the National Health Service (NHS) hospital Trust on the use of chemotherapy in people with small-cell lung cancer. In addition, we have also studied survival in this cohort. As data on radiotherapy are also available in the NLCA, we have also evaluated the impact on survival of radiotherapy use in addition to chemotherapy.

## Materials and Methods

Our data were downloaded from the NLCA and included all patients first seen between January 2004 and 31st December 2008. This dataset has been analysed previously as part of a validation process (Rich *et al*, 2011b) and in terms of the clinical outcomes in patients with non-small cell lung cancer (Rich *et al*, 2011a). For this study, we restricted our analyses to those patients with histologically proven small-cell lung cancer. Our initial dataset included information on sex, age at diagnosis, socioeconomic status (census-derived lower super output area that was linked to the Townsend Index, an area level marker of material deprivation), performance status (as classified by the Eastern Cooperative Oncology Group), stage at presentation (limited or extensive disease) and the NHS Trust, where a patient was first seen and whether chemotherapy had been given. The term NHS Trust refers to the hospital where an individual is first seen in relation to their diagnosis of small-cell lung cancer. The data held by the NLCA on comorbidity were incomplete and are limited to only six disease groups. The audit records only whether or not the presence of this comorbid illness influenced the treatment decision. Therefore, we obtained permission to link this dataset with Hospital Episode Statistics to provide information on in-patient episodes and diagnoses. The Hospital Episode Statistics dataset contains up to 20 diagnoses for each hospital episode coded using International Classification of Diseases (ICD)-10, and our linked dataset covered the 11 financial years between 1997 and 2008. To minimise bias resulting from reverse causation, we ignored the last 3 months of Hospital Episode Statistics data prior to the date of lung cancer diagnosis. We used these data to calculate a composite score of comorbidity, the Charlson Index (Charlson *et al*, 1987), which has been validated in cohorts of men and women with both malignant and non-malignant diseases. The ICD-10 codes for lung cancer were excluded from these calculations. We then divided the cohort into four groups on the basis of their Charlson score. We also used data from Hospital Episode Statistics to provide information on ethnicity.

All NHS Trusts can provide chemotherapy, and so to assess whether there was a range in the provision of chemotherapy across NHS Trusts during our study period, we calculated the proportion receiving chemotherapy in each Trust and then used logistic regression to assess the likelihood of receiving chemotherapy after adjusting for all patient features. We used the largest NHS Trust as the comparator in our regression model, and we included only NHS Trusts that had at least 30 patients with histologically proven small-cell lung cancer to ensure robust estimates.

### Factors affecting receipt of chemotherapy across all National Health Service Trusts

To identify the most important factors associated with an individual's receipt of chemotherapy, we performed logistic regression analyses to assess the likelihood of patients with histologically proven small-cell lung cancer receiving chemotherapy, adjusting for all patient features and clustering on NHS Trust. In this analysis, we also adjusted for a marker of an NHS Trust's participation in clinical trials by estimating whether NHS Trusts were entering a certain proportion of their expected lung cancer patients into clinical trials. To do this, we obtained data from the National Cancer Research Network detailing the number of patients entered into lung cancer clinical trials from each NHS Trust. These data were for the financial year 2008–2009. With data from the national Cancer Registry (2007), we calculated the proportion of expected lung cancer patients being entered into clinical trials at each NHS Trust. To allow inclusion in our multivariate model, we created a binary variable for participation in clinical trials (low versus high participation), by using a cutoff at 5% of expected patients being entered into trials. This level was chosen because it was above the mean proportion of involvement in clinical trials for all NHS Trusts, but was still an achievable target as approximately a third of all patients with small-cell lung cancer where seen in centres with high trial participation. We also tried to quantify lung cancer Multi-Disciplinary Team (MDT) performance by using the results of the Peer Review process 2004–2007. The MDT is the team of clinicians and nurse specialists involved in the diagnostic and therapeutic management of patients with lung cancer. It can include respiratory physicians, radiologists, oncologists, thoracic surgeons, histopathologists and palliative care physicians. We took the overall score for each NHS Trust, and defined as excellent any NHS Trust that was in the top quartile, thus creating a binary variable. But this was subsequently dropped from multivariate regression analyses because of the lack of evidence to support the assumption that it influenced access to chemotherapy or overall survival.

### Survival related to the receipt of chemotherapy

For our survival analyses, we created a ‘start’ date using the date of diagnosis where available. In the absence of this, we calculated a surrogate ‘date of diagnosis’ using the date of first clinic appointment, and, based on the median interval between these dates for the cohort overall (10 days), we interpolated a surrogate ‘start’ date for all those without a ‘date of diagnosis’. The end date was either the patient's date of death (obtained from the Patient Demographics Service) or the date the dataset was downloaded, which was 30 September 2009. Because our objective was to assess the effect of chemotherapy on survival, patients with a date of death the same as, or earlier than, the date of diagnosis were excluded from our survival analyses. We performed Cox regression analyses to calculate hazard ratios for overall mortality in patients receiving chemotherapy compared with those receiving no treatment and then constructed a multivariate model mutually to adjust for all patient features and NHS Trust trial involvement. The final Cox regression model included clustering by NHS Trusts. We then restricted this multivariate Cox regression model to include only patients who had received chemotherapy, to assess whether chemoradiotherapy conferred any survival advantage over chemotherapy alone. We checked the proportional hazards assumption for our models by inspecting Nelson–Aalen plots.

Finally, to determine whether patients first seen at a centre with high trial participation were different from those first seen in a centre with low trial participation, we compared the demographic features of patients between these two groups of NHS Trusts. For the subgroup of patients who had received chemotherapy, we used a Cox regression model to assess survival according to whether a patient had been first seen in a centre with high compared with low trial participation, adjusting for all patient features and clustering by NHS Trust.

## Results

Our dataset contained a total of 87 252 patients who were first seen at an English NHS Trust between January 2004 and 31 December 2008. We excluded 6286 patients (7%) because there were missing data for the NHS Trust where the patient had first been seen. There were 7845 (10%) patients with histologically proven small-cell lung cancer of whom 54% were men, and the median age of these patients was 69 years (interquartile range 62–76 years), 2 years younger than for the cohort overall. In total, 1781 patients (23%) had evidence of comorbid disease with a Charlson score of 4 or more, compared with 19% of the cohort overall. There were 44 NHS Trusts with >5% of expected lung cancer patients being entered into clinical trials, henceforth called centres with high trial participation. Of the 7845 patients with histologically proven small-cell lung cancer, 2524 (32%) were first seen in centres with high trial participation, which was a similar proportion to the cohort overall (31%).

### Variation in chemotherapy use across National Health Service Trusts

Our analysis of the use of chemotherapy at each NHS Trust in England showed wide variation. In the NHS Trusts with more than 30 patients, the overall proportion receiving chemotherapy was 0.61, the same as for the whole group with small-cell lung cancer. The actual proportion ranged from 0.14 to 0.86 at individual NHS Trusts (interquartile range 0.53–0.71). Adjusting for all patient features, there was significant variation (*P*<0.001) in the odds ratios for receiving chemotherapy in the same group of NHS Trusts, with the largest Trust as comparator. The individual NHS Trust level odds ratios ranged from 0.03 (95% confidence interval (CI) 0.014, 0.07) to 4.47 (95% CI 1.46, 13.72), with an interquartile range of 0.42–1.02.

### Receipt of chemotherapy

A total of 4820 (61%) patients with histologically proven small-cell lung cancer received chemotherapy, of whom 861 (18%) also received radiotherapy. Table 1 shows the results of logistic regression analyses of likelihood of receiving chemotherapy. Age at diagnosis, performance status, stage and comorbidity all showed important independent associations with receipt of chemotherapy. As age increased, the likelihood of receiving chemotherapy decreased, with an odds ratio of 0.74 (95% CI 0.64, 0.86) in the second quintile (63–69 years) and an odds ratio of 0.59 (95% CI 0.50, 0.69) in the third quintile (70–75 years) compared with the youngest group. Patients with a performance status of 2 were less likely to receive chemotherapy compared with patients with a performance status of 0 (adjusted odds ratio 0.58, 95% CI 0.45, 0.74). Extensive stage disease at diagnosis was associated with a reduction in the likelihood of receiving chemotherapy compared with those patients with limited disease (adjusted odds ratio 0.61, 95% CI 0.47, 0.78). A Charlson index of 4 or more was associated with a reduced likelihood of receiving chemotherapy compared with a Charlson index of 0 (adjusted odds ratio 0.50, 95% CI 0.42, 0.58). Sex, ethnicity and socioeconomic status were not associated with access to chemotherapy.

If a patient was first seen in an NHS Trust defined as a centre with high trial participation, they were more likely to receive chemotherapy than those at a centre with low trial participation, even after adjusting for all patient features (adjusted odds ratio 1.42, 95% CI 1.06, 1.90). When we performed a restricted analysis with only those patients without missing data (*N*=3059), the results were very similar (adjusted odds ratio for centres with high *vs* low trial participation 1.50, 95% CI 1.03, 2.16).

### Survival analysis

A small number of patients (63) had a date of death on or before the date of diagnosis, and therefore were excluded from the survival analyses. The median survival for the remaining cohort of 7782 patients with histologically proven small-cell lung cancer was 182 days (interquartile range 44–368 days). Table 2 shows the results of univariate and multivariate Cox regression analyses, and demonstrates that women had a better prognosis than men. As age, stage, performance status and comorbidity increased, prognosis worsened. The adjusted hazard ratio for patients with a Charlson index of 4 or more was 1.58 (95% CI 1.42, 1.74) compared with those patients with a Charlson index of 0. Socioeconomic status and ethnicity had no effect on overall survival. Whether the NHS Trust where a patient was first seen was a centre with high trial participation or not did not affect overall survival (adjusted hazard ratio 0.99, 95% CI 0.88, 1.10). There was no evidence that our proportional hazard assumption was not met.

**Figure 1:**
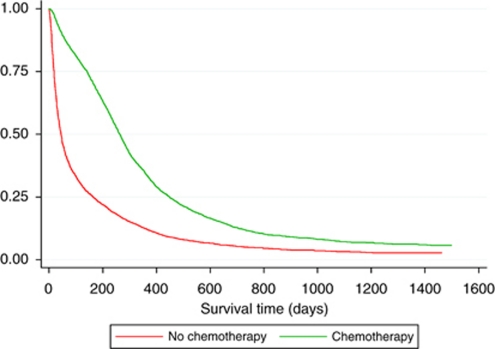
Kaplan–Meier survival curve for patients with proven small-cell lung cancer based on receipt of chemotherapy (*N*=7782).

Table 2 also shows that patients who received chemotherapy had a lower mortality compared with those who did not, in spite of adjusting for all patient features (adjusted hazard ratio 0.51, 95% CI 0.46, 0.56). When we performed a restricted analysis with only those patients without missing data (*N*=3059), the results were very similar (adjusted hazard ratio for yes *vs* no chemotherapy 0.49, 95% CI 0.41, 0.58). The survival of patients over time who did and did not receive chemotherapy is shown in Figure 1. In the subgroup of patients with limited disease (1319 patients) where 78% received chemotherapy, there was a lower overall mortality rate compared with those who did not receive chemotherapy (adjusted hazard ratio 0.62, 95% CI 0.50, 0.76). The median survival for patients with limited stage disease who received chemotherapy was 399 days (interquartile range 241–686 days), compared with a median survival of just 139 days (interquartile range 37–381 days) in those who did not receive chemotherapy. Table 3 demonstrates that those patients with limited stage disease who received chemoradiotherapy had a better overall survival than those who received chemotherapy alone (adjusted hazard ratio 0.72, 95% CI 0.62, 0.84).

The demographic features of patients first seen in centres with high and low trial participation were similar (Table 4), although the proportion of patients from the least affluent quintile of society was higher in centres with high compared with low trial participation. Although there were differences in stage and performance status between the two types of centres, this will in part reflect the size of the cohort. The main difference between the high and low trial participation centres were in the missing data. Most importantly in the group of patients likely to receive chemotherapy, good performance status (0–1) and limited stage disease, the proportions were very similar (36% and 37% and 16% and 17%, respectively, between high and low centres). Of the 4820 (61%) patients who received chemotherapy, 34% were first seen in centres with high trial participation. Survival after chemotherapy was not affected by whether or not a patient had been first seen in a centre with high compared with low trial participation, adjusted hazard ratio 1.05 (95% CI 0.97, 1.13).

## Discussion

### Principle findings

Our results demonstrate that there is considerable variation in the use of chemotherapy in people with small-cell lung cancer. Older age and the presence of comorbidity were both associated with a decrease in the use of chemotherapy, but even after allowing for these there was wide variations in use between NHS Trusts in England. Trusts with an interest in recruiting people into lung cancer clinical trials in general were more likely to give chemotherapy to people with small-cell lung cancer, and this difference was not explained by individual patient features.

In our study, male sex, increasing age, comorbidity, worsening performance status and extensive stage disease were all independently associated with a worse survival. Whether or not a patient received chemotherapy was also independently associated with survival (adjusted hazard ratio of 0.51, 95% CI 0.46, 0.56). The beneficial effects of chemotherapy on survival among the people who got chemotherapy were the same whether a patient was first seen in a high or low trial centre, suggesting that the increased use of chemotherapy in high trial centres was not associated with an increase in chemotherapy-related deaths. This in turn suggests that the high trial centres are not tending to over treat people and that there is scope to increase the use of chemotherapy in the low trial centres.

### Strengths and weaknesses

Although the NLCA is non-mandatory, we have previously shown that this is a valid and representative dataset (Rich *et al*, 2011b). There is also evidence that the case ascertainment rate in the NLCA is now in excess of 90% (National Lung Cancer Audit, 2008, 2009), and thus this study has used one of the largest contemporary, clinical lung cancer datasets in the world. One potential weakness is that our data on comorbidity relate only to diagnoses associated with hospital admissions. As a result, we may not have captured details of every condition managed independently by general practitioners, and thus our derived Charlson indices may be too low, and there may be some residual confounding by comorbidity. However, we think that this is unlikely to be the case, as the range of Charlson indices observed in our cohort is similar to those in cohorts of patients from a general practitioner dataset (Khan *et al*, 2010) and patients with lung cancer (Wang *et al*, 2007; Asmis *et al*, 2008). Furthermore, our analyses showed that although comorbidity was an important predictor of survival, it did not confound the association between the use of chemotherapy and survival.

We acknowledge that using entry into clinical trials as a surrogate for chemotherapy practice may in itself explain the variation in access to chemotherapy described. However, the cutoff for our high trial centres was only 5% entry of expected patients into clinical trials, and thus the majority of individuals with small-cell lung cancer would have received chemotherapy outside a clinical trial. Furthermore, this study analyses the extent of variation among NHS Trusts having accounted for all patient features.

It is not possible to elicit from the dataset the number of patients who were offered chemotherapy but declined, nor the frequency of side-effects and toxicity from the chemotherapy.

### Comparison with other studies

The annual reports from the NLCA have described variation in chemotherapy use among individuals with small-cell lung cancer across England, although they have not adjusted for comorbidity. In the 2009 report (which assessed data from patients first seen in 2008), this proportion ranged from 0.00 to 1.00, which shows that the variation over the years 2004–2008 that we have found in this study still holds at the end of the study period. In a separate study, Jack *et al* (2003) described variation in treatment rates and overall survival in lung cancer patients in South East England, but again no adjustment was made for performance status or comorbidity. Patients first seen at a radiotherapy centre were more likely to receive ‘active treatment’, chemotherapy and radiotherapy (Jack *et al*, 2003). Several major policy documents have been published by the Department of Health over the past 15 years (Department of Health, 1995, 2000, 2007). One of the major themes has been the creation of specialist cancer centres, and there is evidence that patients first seen by a lung cancer specialist are more likely to receive ‘active treatment’, including chemotherapy, than those who are not (Jack *et al*, 2006), and centralised referral for lung cancer has been associated with improved survival rates (Stiller, 1994). However, the creation of specialist cancer centres will potentially generate greater inequality in access to treatment as the distance and time spent travelling increases. Jones *et al* (2008) and Crawford *et al*, (2009) have both described a reduction in the likelihood of receiving chemotherapy in lung cancer patients as distance to hospital increased, and Campbell *et al* (2000) reported a poorer survival after diagnosis for individuals with lung cancer as distance from a cancer centre increased. Given chemotherapy is available in all NHS Trusts, and recommended for the treatment of all patients with small-cell lung cancer (Department of Health, 1998; National Institute for Clinical Excellence, 2005), it should be possible to make access to this treatment fairer. Our results have shown that the increased use of chemotherapy in high trial centres is not at the detriment of overall patient survival. Therefore, there is reason to expect that increasing the rate of chemotherapy use in small-cell lung cancer would result in patient benefit.

We have also been able to demonstrate in a large cohort that chemoradiotherapy has a survival advantage over chemotherapy alone. This supports the previously reported long-term survival gain of this multimodality treatment (Pignon *et al*, 1992; Warde and Payne, 1992), and would suggest that chemoradiotherapy becomes the treatment of choice in individuals with limited stage small-cell lung cancer.

Our research also showed that as age increases, the use of chemotherapy decreases, even after adjusting for stage, performance status and comorbidity. This is in keeping with several publications (Brown *et al*, 1996; Coebergh *et al*, 1999; Ludbrook *et al*, 2003), despite evidence that overall response to chemotherapy is not diminished in people with small-cell lung cancer aged over 70 years (Li *et al*, 2009). Janssen-Heijnen *et al* (1998) found that in patients over the age of 70 years the presence of even a single comorbid illness reduced the use of chemotherapy, suggesting a reluctance to use these treatments in older people (Janssen-Heijnen *et al*, 1998).This supports our evidence that it is not the associated comorbidity rise with age that is wholly responsible for the observed decline in chemotherapy use as patients get older. The apparent reluctance to provide chemotherapy in elderly patients with small-cell lung cancer is not supported by evidence of a poor safety record (Smit *et al*, 1989; Ludbrook *et al*, 2003; Yau *et al*, 2006).

### Implications of this study

Our results have shown evidence of the beneficial effects of chemotherapy for people with small-cell lung cancer in England, but also the evidence of variations in access to this treatment dependent upon age and hospital attended. The main determinants of Trust level variation are not known, and this is an important research question that needs addressing in the future development of the NLCA. The standards set in the 2004–2007 Peer Review process do not appear to have captured sufficient detail to distinguish between the performances of multi-disciplinary teams in different NHS Trusts. With regard to age, it is clear that further debate is needed in the lung cancer community about the decision to withhold treatment from older people with lung cancer.

## Figures and Tables

**Table 1:** Logistic regression analysis of patient features and NHS Trust trial entry on the likelihood of receiving chemotherapy for proven small-cell lung cancer (clustered by NHS Trust)

	**Total patients (*N*=7845)**	**Patients receiving chemotherapy (*N*=4820)**	**%^a^**	**Unadjusted OR**	**Adjusted OR** b	***P*-value** c
*Sex*						
Male	4245	2560	60			0.106
Female	3600	2260	63	1.11 (1.01, 1.22)	1.08 (0.98, 1.19)	
						
*Age quintile* (years)						
1 (30–62)	2174	1616	74			<0.001
2 (63–69)	1928	1292	67	0.70 (0.61, 0.80)	0.74 (0.64, 0.86)	
3 (70–75)	1771	1079	61	0.54 (0.47, 0.62)	0.59 (0.50, 0.69)	
4 (76–80)	1170	580	50	0.34 (0.29, 0.39)	0.39 (0.32, 0.47)	
5 (81–101)	802	253	32	0.16 (0.13, 0.19)	0.19 (0.15, 0.24)	
						
*Performance Status*						
PS 0	977	779	80			<0.001
PS 1	1925	1504	78	0.91 (0.75, 1.10)	1.08 (0.87, 1.35)	
PS 2	1444	901	62	0.42 (0.35, 0.51)	0.58 (0.45, 0.74)	
PS 3	876	341	39	0.16 (0.13, 0.20)	0.25 (0.19, 0.33)	
PS 4	284	30	11	0.03 (0.02, 0.05)	0.05 (0.03, 0.07)	
Missing	2339	1265	54	0.30 (0.25, 0.36)	0.42 (0.32, 0.55)	
						
*Stage*						
Limited	1323	1025	77			<0.001
Extensive	3078	1873	61	0.45 (0.39, 0.52)	0.61 (0.47, 0.78)	
Missing	3444	1922	56	0.37 (0.32, 0.42)	0.45 (0.34, 0.59)	
						
*Townsend quintile*						
1 (Most affluent)	1087	675	62			0.075
2	1385	876	63	1.05 (0.89, 1.24)	1.02 (0.85, 1.24)	
3	1530	922	60	0.93 (0.79, 1.09)	0.92 (0.77, 1.11)	
4	1669	1008	60	0.93 (0.80, 1.09)	0.87 (0.72, 1.09)	
5 (Least affluent)	2154	1327	62	0.98 (0.84, 1.14)	0.85 (0.67, 1.08)	
Missing	20	12	60	0.92 (0.37, 2.26)	0.65 (0.25, 1.87)	
						
*Ethnic group*						
White	6061	3739	62			0.107
Black	31	16	52	0.66 (0.33, 1.34)	0.38 (0.11, 1.29)	
Asian	399	240	60	0.94 (0.76, 1.15)	1.02 (0.80, 1.27)	
Mixed	14	10	71	1.55 (0.49, 4.96)	1.75 (0.58, 5.32)	
Other	38	20	53	0.69 (0.36, 1.31)	0.60 (0.34, 1.10)	
Missing	1302	795	61	0.97 (0.86, 1.10)	0.94 (0.81, 1.09)	
						
*Charlson index*						
0	3482	2441	70			<0.001
1	1492	904	61	0.66 (0.58, 0.74)	0.79 (0.69, 0.91)	
2 or 3	1090	625	57	0.57 (0.50, 0.66)	0.76 (0.65, 0.90)	
4+	1781	850	48	0.39 (0.35, 0.44)	0.50 (0.42, 0.58)	
						
*Trial entry* (%)						
<5	5321	3162	59			0.017
>5	2524	1658	66	1.31 (1,18, 1.44)	1.42 (1.06, 1.90)	

Abbreviations: NHS=National Health Service; OR=odds ratio.

a Percentage of each variable who received chemotherapy.

b OR for chemotherapy adjusted for all other variables in the table. Analysis clustered by the NHS Trust.

c All non-binary variables are tested for trend except ethnicity, which is a likelihood ratio test.

*N*=number of patients within each variable who received chemotherapy.

All missing values were removed prior to the calculation of probability.

**Table 2:** Cox regression analysis of patient features, NHS Trust trial entry and the patient's receipt of chemotherapy on overall survival

	**Total patients (*N*=7782)**	**Deaths (*N*=6981)**	**%^a^**	**Unadjusted HR**	**Adjusted HR^b^**	***P*-value** c
*Sex*						
Male	4206	3838	91			<0.001
Female	3576	3143	88	0.84 (0.80, 0.88)	0.86 (0.82, 0.90)	
						
*Age quintile* (years)						
1 (30–62)	2161	1859	86			<0.001
2 (63–69)	1917	1731	90	1.22 (1.14, 1.30)	1.12 (1.04, 1.21)	
3 (70–75)	1757	1561	89	1.31 (1.23, 1.40)	1.20 (1.11, 1.30)	
4 (76–80)	1159	1079	93	1.62 (1.51, 1.75)	1.31 (1.19, 1.44)	
5 (81–101)	788	751	95	2.07 (1.90, 2.25)	1.47 (1.32, 1.64)	
						
*Performance status*						
PS 0	975	772	79			<0.001
PS 1	1919	1653	86	1.39 (1.28, 1.52)	1.34 (1.24, 1.45)	
PS 2	1437	1344	94	2.19 (2.01, 2.40)	1.83 (1.67, 2.00)	
PS 3	868	847	98	3.82 (3.46, 4.21)	2.65 (2.36, 2.99)	
PS 4	269	265	99	8.63 (7.40, 9.95)	5.01 (4.05, 6.19)	
Missing	2314	2100	91	1.86 (1.71, 2.02)	1.63 (1.50, 1.77)	
						
*Stage*						
Limited	1319	1043	79			0.001
Extensive	3053	2894	95	2.45 (2.28, 2.63)	2.07 (1.92, 2.25)	
Missing	3410	3044	89	1.74 (1.62, 1.87)	1.43 (1.31, 1.57)	
						
*Townsend quintile*						
1 (Most affluent)	1075	947	88			0.341
2	1378	1234	90	1.04 (0.96, 1.13)	1.07 (0.98, 1.17)	
3	1523	1365	90	1.08 (0.99, 1.17)	1.07 (0.97, 1.17)	
4	1650	1490	90	1.05 (0.97, 1.14)	1.07 (0.98, 1.18)	
5 (Least affluent)	2138	1929	90	1.05 (0.97, 1.13)	1.07 (0.96, 1.19)	
Missing	18	16	89	1.13 (0.69, 1.85)	1.52 (1.15, 2.02)	
						
*Ethnic group*						
White	6015	5439	90			0.422
Black	31	26	84	0.71 (0.49, 1.05)	0.79 (0.56, 1.11)	
Asian	396	344	87	1.06 (0.95, 1.19)	1.01 (0.88, 1.15)	
Mixed	14	11	79	0.69 (0.38, 1.24)	0.78 (0.47, 1.31)	
Other	37	32	87	1.00 (0.71, 1.42)	0.84 (0.46, 1.52)	
Missing	1289	1129	88	1.00 (0.93, 1.06)	1.04 (0.94, 1.14)	
						
*Charlson index*						
0	3466	3015	87			<0.001
1	1483	1301	88	1.23 (1.15, 1.32)	1.07 (1.01, 1.14)	
2 or 3	1080	967	90	1.35 (1.25, 1.45)	1.11 (1.02, 1.22)	
4+	1753	1698	97	2.09 (1.97, 2.22)	1.62 (1.49, 1.77)	
						
*Chemotherapy*						
No	2967	2825	95			<0.001
Yes	4815	4156	86	0.43 (0.41, 0.45)	0.51 (0.46, 0.56)	
						
*Trial entry* (%)						
<5	5282	4739	90			0.83
>5	2500	2242	90	0.96 (0.91, 1.01)	0.99 (0.88, 1.10)	

Abbreviations: HR=hazard ratio; NHS=National Health Service.

a Percentage of patients from each subgroup who have died.

b HR adjusted for all other variables in the table. Analysis clustered by the NHS Trust features.

c All non-binary variables are tested for trend except ethnicity, which is a likelihood ratio test.

All missing values were removed prior to the calculation of probability.

A total of 63 patients had a date of diagnosis on or before their date of death, and were excluded from survival analyses (*N*=7782).

**Table 3:** Cox regression analyses assessing survival in patients with small-cell lung cancer who received chemoradiotherapy compared with those receiving chemotherapy alone

	***N***	***N* who died**	**%^a^**	**Unadjusted HR (95% CI)**	**Adjusted HR (95% CI)^b^**	***P*-value**
*Whole cohort*	7782					
CTx alone	3914	3463	88			<0.001
CTx and RTx	861	670	78	0.66 (0.61, 0.72)	0.70 (0.65, 0.76)	
*Limited stage*	1319					
CTx alone	737	594	81			<0.001
CTx and RTx	280	184	66	0.69 (0.58, 0.81)	0.72 (0.62, 0.84)	

Abbreviations: CI=confidence interval; CTx=chemotherapy; HR=hazard ratio; *N*=number; NHS=National Health Service; RTx=radiotherapy.

a Percentage of patients who died.

b HR adjusted for sex, age, performance status, stage (whole cohort only), Townsend quintile, ethnic group and Charlson index. Clustered on NHS Trust.

Some patients had no record of any treatment received, and some received surgery, whereas others received radiotherapy alone.

Whole cohort; no treatment, *N*=2360; surgery, *N*=148; and radiotherapy alone, *N*=499.

Limited stage only: no treatment, *N*=218; surgery, *N*=20; and radiotherapy alone, *N*=64.

**Table 4:** Demographic features of patients with small-cell lung cancer based on where they are first seen

	**Centre with high trial participation**		**Centre with low trial participation**		
	**(*N***=**2524)**	**%^a^**	**(*N*=5321)**	**%^a^**	***P*-value**
*Sex*					0.446
Male	1401	56	2844	53	
Female	1123	44	2477	47	
					
Median age	69 years (IQR 61–75 years)		69 years (IQR 62–76 years)		
					
*Performance status*					0.001
0	331	13	646	12	
1	579	23	1346	25	
2	420	17	1024	19	
3	254	10	622	12	
4	74	3	210	4	
Missing	866	34	1473	28	
*Stage*					0.001
Limited	393	16	930	17	
Extensive	844	33	2234	42	
Missing	1287	51	2157	41	
*Charlson index*					0.175
0	1124	46	2358	44	
1	460	18	1032	19	
2 or 3	350	14	740	14	
4+	590	23	1191	22	
*Townsend quintile*					<0.001
1 (Most affluent)	351	14	736	14	
2	406	16	979	18	
3	460	18	1070	20	
4	483	19	1186	22	
5 (Least affluent)	821	33	1333	25	
*Chemotherapy*					<0.001
No	866	34	2159	41	
Yes	1658	66	3162	59	

Abbreviation: IQR=interquartile.

a Percentage.

A centre with high trial participation was one that entered 5% or more its expected lung cancer patients into lung cancer clinical trials.

Total number with small-cell lung cancer is 7845.
